# Results from a Cross-Sectional Observational Study Examining Irritable Bowel Syndrome Patients Six Months After Finishing Their Participation in the ViIBS Trial

**DOI:** 10.3390/nu16223911

**Published:** 2024-11-15

**Authors:** Henning Sommermeyer, Krzysztof Chmielowiec, Malgorzata Bernatek, Pawel Olszewski, Jaroslaw Kopczynski, Jacek Piątek

**Affiliations:** 1 Department of Health Sciences, Calisia University, Nowy Swiat 4, 62-800 Kalisz, Poland; drpiatek@o2.pl (M.B.); pawel.r.olszewski@gmail.com (P.O.); jkopczynski106@gmail.com (J.K.); drpiatek@interia.eu (J.P.); 2 Department of Hygiene and Epidemiology, Collegium Medicum, University of Zielona Góra, 28 Zyty St., 65-046 Zielona Góra, Poland; k.chmielowiec@inz.uz.zgora.pl

**Keywords:** gut microbiota, probiotic bacteria, symbiotic, irritable bowel syndrome, ViIBS trial

## Abstract

Background/Objectives. A recent clinical (ViIBS) trial investigating the effects of a balanced multi-strain synbiotic in irritable bowel syndrome (IBS) patients showed that twelve weeks of treatment resulted in significant improvements across all major IBS symptoms. The current observational study pursued three aims: investigate patients’ attitude towards the intake of pro- or synbiotics during the six months after finishing their trial participation, determine the severity of IBS symptoms, and assess IBS diagnosis scores. Methods. During a single six-month follow-up examination, patients were asked about the intake of probiotics or synbiotics. For the study, former placebo-group patients who abstained from taking probiotics were compared with synbiotic-group patients who continued taking the tested synbiotic. IBS symptom severity was assessed with the IBS—Severity of Symptoms Scale and the IBS diagnosis score with the IBS questionnaire of the World Gastroenterology Organisation. Results. The control group comprised 17 patients (out of 70 from the placebo group participating in the follow-up) and the treatment group 75 (out of 91 examined). IBS symptom severity was significantly lower in the treatment group (23.5 ± 33.1) than in the placebo group (232.6 ± 35.1). IBS diagnosis scores were 5.9 ± 2.5 and 21.2 ± 2.0 in the treatment and control group, respectively. Conclusions. Measurement values for the treatment group indicate the absence of IBS. The results indicate that the prolonged administration of the balanced multi-strain synbiotic can potentially reduce IBS symptom severity and IBS diagnosis scores to levels indicating the absence of IBS, an observation to be followed up in a controlled clinical trial.

## 1. Introduction

Irritable bowel syndrome (IBS) is a functional disorder of the gastrointestinal system which affects about one in ten of the general population [1]. Patients with IBS suffer from recurrent abdominal pain, bloating, and changes in stool form and frequency [2]. The annoying and volatile nature of the main IBS symptoms impairs the quality of life and the work productivity of IBS patients [3,4]. The management of the IBS patient causes significant resource utilization and costs for healthcare systems [5,6]. While the etiology of IBS remains not fully understood, a disturbed gut microbiota is considered to be a major contributing factor [7,8,9,10].

In recent years, the therapeutic effects of products containing probiotic bacteria have been evaluated in a number of clinical trials with IBS patients [11,12,13,14,15]. Among the preparations tested were products from the category of probiotics and synbiotics. Probiotics are products containing one (mono-strain) or several (multi-strain) probiotic bacteria [16]. Synbiotics contain prebiotic ingredients (e.g., fructo-oligosaccharides) in addition to the bacterial component, which serve the bacteria as a source of energy [17]. Balanced multi-strain products are characterized by a bacteria mixture which is not quantitatively dominated by one or more bacterial strains.

Our group recently published results from the ViIBS trial, a multi-center, randomized, double-blind, placebo-controlled clinical trial conducted in Poland. The study evaluated the effects of a balanced multi-strain synbiotic in primary care patients with moderate to severe IBS [18]. This synbiotic has been marketed as a food supplement for many years in certain countries (Germany and Singapore). Patients need no prescription when purchasing the product; however, it is not covered by their health insurance. The diagnosis of the patients participating in the trial was performed using the IBS questionnaire for HCPs of the World Gastroenterology Organisation [19]. Treatment effects were evaluated over a period of 12 weeks. Compared to patients from the placebo group, patients taking the synbiotic experienced, among other treatment-related effects, (i) a significant reduction in IBS symptom severity measured with the IBS—Severity of Symptoms Scale (IBS-SSS) [20], (ii) improvements in global IBS symptoms assessed with the IBS—Global Improvement Scale (IBS-GIS) [21], and (iii) adequate relief determined by using the IBS–Adequate Relief (IBS-AR) scale [22].

After the trial ended, participating patients were invited for a follow-up examination scheduled six months later. At the time of the invitation, patients were not informed whether they had been part of the placebo or synbiotic treatment group of the ViIBS trial, as the trial data were unblinded at a later date. However, they were given the name of the synbiotic product investigated in the trial. Since the product is not commercially available in Poland, patients were informed that they could order it from an Internet pharmacy in Germany that fulfills international orders. Between the end of the trial and the follow-up examination, patients were free to use products containing probiotic bacteria.

The key objectives of this cross-sectional observational study were as follows: (i) finding out how many patients would show up for an examination after 6 months; (ii) collecting information about patients’ attitude to continuing to take the tested product or other products containing probiotic bacteria after their trial experience; (iii) measuring the severity of IBS symptoms using the IBS-SSS six months after the patient had finished their ViIBS trial participation, especially for those patients who stopped and those who continued to take the tested synbiotic; and (iv) repeating the IBS diagnosis using the IBS questionnaire for HCPs of the World Gastroenterology Organisation, which had already been used at the beginning of the controlled trial.

Cross-sectional observational studies have a number of inherent limitations, among which is a susceptibility to bias, which restricts their ability to determine causality [23]. However, at the same time, they are inexpensive and easy to conduct, and can provide preliminary insights which can be helpful for planning future randomized clinical trials [24]. We had limited expectations for collecting data from a large number of patients, especially those who might have continued taking the synbiotic tested in the ViIBS trial. Major concerns were (i) the length of time (six months) between finishing the trial participation and the examination and (ii) the fact that the tested multi-strain synbiotic is not easily commercially available for patients in Poland. However, being able to collect data even from a limited number of patients was considered useful for the planning of a follow-up to the ViIBS trial.

## 2. Materials and Methods

### 2.1. Study Design

During the final examination, all patients were invited for a follow-up examination six months (180 days) after they had finished their participation in the ViIBS trial. In line with a purely observational trial design, patients were informed that, during the 180-day period, they were free to choose whether or not to take products containing probiotic bacteria (e.g., Vivatlac^®^ Synbiotic). If they decided to take such products, patients were asked to record which product(s) and for how many days they had taken them. The study protocol was approved by the Ethics Committee of Calisia University (project identification code 1/2023, 25 January 2023).

### 2.2. Basic Characteristics of Study Participants

The basic characteristics of the patients included in the analysis were determined from the data collected during the enrollment examination of the ViIBS trial. Patients included in the study were aged 18 to 65 years, diagnosed with IBS according to the IBS questionnaire for healthcare providers (HCPs) and had an IBS severity of symptoms score assessed with the IBS-SSS of at least 175 points, representing moderate to severe cases of IBS.

### 2.3. Balanced Multi-Strain Synbiotic

**Figure 1:**
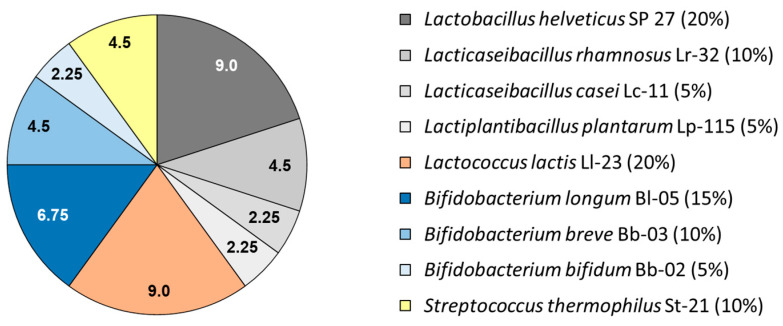
Composition of the balanced nine-strain synbiotic. Figures in the pie chart represent number of CFUs (times 10^8^) of the respective probiotic strain per capsule. Percentages given in the legend indicate percent contribution of the respective strain to the total amount of CFUs per capsule. In addition to the probiotic bacteria, each capsule contains 63 mg of fructo-oligosaccharides as a prebiotic component. The capsule is an enteric-coated hydroxypropyl methylcellulose capsule.

The balanced multi-strain synbiotic (Vivatlac^®^ Synbiotic) taken by patients is an enteric-coated capsule preparation with each capsule containing a total of 4.5 × 10^9^ colony-forming units (CFUs) of nine different probiotic bacteria strains. The balanced composition of the mixture of probiotic bacteria is shown in Figure 1. A special enteric coating protects the bacteria of the mixture against inactivation in the upper part of the gastrointestinal tract [25]. The synbiotic has been commercially available in Germany (Vivatlac^®^ Synbiotikum, Vivatrex^®^ GmbH, Rees, Germany) for many years and was purchased for the trial.

### 2.4. Data Collection

Examinations of patients were performed at the family doctor’s clinic, in Stawiszyn, West Poland, and the family doctor’s outpatient clinic “Panacea”, in Krynki, East Poland, by a total of eight physicians. The first patients were examined in December 2023 and the last examination took place in May 2024. Examination results were reported by study doctors using a medical data logbook. In the logbook, the physicians noted the date of examination, the patients’ name, birth date, and ViIBS trial participation number. Patients were asked if they had taken pro- or synbiotics since finishing their participation in the ViIBS trial (with predefined answers: yes or no). If they answered yes, they were asked which products they had taken, with a free text field in the doctor’s data logbook to note the brand names. The next question inquired how many days the patient had taken the product over the last 180 days, with options to select from the following categories: 1–30, 31–90, or 91–180 days.

### 2.5. Study Endpoints

After patients answered the questions about their intake of probiotic bacteria products, study physicians assessed the severity of IBS symptoms by using the IBS-SSS [20]. The IBS-SSS is a visual analog scale comprising five questions assessing five major aspects of the IBS symptoms (severity and frequency of abdominal pain, severity of abdominal flatulence, dissatisfaction with bowel habit, and interference with quality of life). A detailed description of the scale can be found in the publication of the ViIBS trial [18]. Severity of IBS can be graded based on the total score measured with the IBS-SSS. Severe IBS is assumed in patients with IBS-SSS scores above 300, while those with scores between 175 to 300 are considered as moderate, those with 75 to 175 as mild, and those with less than 75 as non-IBS patients, respectively.

After determining the severity of IBS symptoms, patients underwent an examination using the IBS questionnaire for health care providers of the World Gastroenterology Organisation (WGO) [19].

### 2.6. Statistical Analyses

Data for weight, height, body mass index, age, IBS-SSS score, and diagnostic score obtained with the IBS questionnaire for HCPs in both groups were tested for normal distribution using the Kolmogorov–Smirnov normality test. As the data did not meet the assumption of normal distribution, and the sample sizes between groups were unequal, non-parametric statistical tests were employed. The Mann–Whitney U test was used to compare quantitative variables, including weight, height, body mass index, age, IBS questionnaire for HCPs scores, and the IBS-SSS scores between the control and the treatment group at different examination time points. Gender, IBS severity, and IBS stool type distribution were tested using the Pearson’s Chi-squared test to evaluate the associations between categorical variables across groups. The homogeneity of variances of the IBS diagnostic scores obtained with the IBS questionnaire for HCPs for the study groups at enrollment and at the end of the 6-month follow-up period was assessed using Levene’s test. Due to the lack of normal distribution and non-homogeneity of variances, Kruskal–Wallis test with Dunn’s multiple comparisons was applied to determine the differences in IBS questionnaire scores among groups, as it is suitable for non-normally distributed data across more than two groups. All calculations were performed using Statistica software version 14.1.0.4 (Tibco Software Inc., Palo Alto, CA, USA). The results were considered statistically significant at *p*-value ˂0.05.

## 3. Results

### 3.1. Follow-Up Rates, Patient Flow, and Baseline Characteristics of the Two Patient Groups

**Figure 2:**
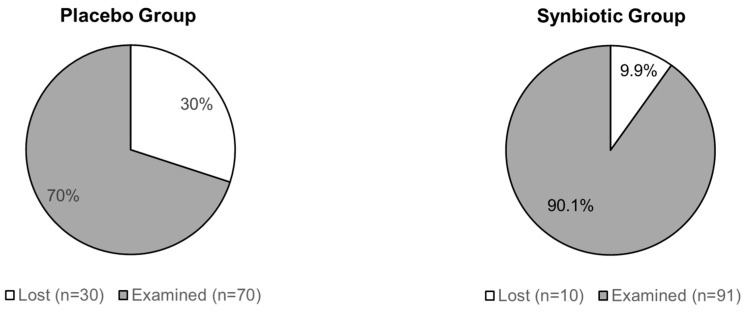
Follow-up rates of patients from the placebo and the synbiotic treatment groups six months after finishing their participation in the ViIBS trial.

All 201 patients who completed the ViIBS trial were invited for a follow-up examination 6 months after finishing their trial participation. A total of 161 patients (80.1%) took up this invitation and were examined. Follow-up rates were 70% (*n* = 70) for patients from the placebo group and 90.1% (*n* = 91) for patients from the synbiotic treatment group (Figure 2). Follow-up examinations were performed between 180 to 189 days (average 185.4 days) after the last examination of the ViIBS trial with no difference between the two patient groups evaluated.

### 3.2. Patient Flow

**Figure 3:**
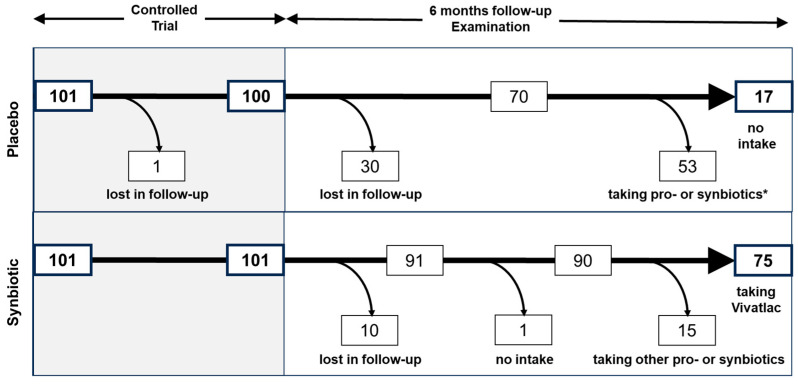
Patient flow of patients from the placebo and the synbiotic treatment groups through the ViIBS trial and the six-month follow-up observational study. * Of the 53 patients of the ViIBS trial placebo group declared they had taken a product containing probiotic bacteria, 25 took Vivatlac synbiotic, the preparation evaluated in the trial.

Out of the 70 patients from the placebo group who were examined, 53 (75.7%) declared that they had taken products containing probiotic bacteria during the 6 months after finishing the trial (Figure 3). These patients were excluded from further analysis for the present study. The remaining 17 patients stated that they abstained from taking any kind of product containing probiotic bacteria. These patients were defined as the “control group” for the present study.

Of the 91 patients from the synbiotic treatment group, only 1 patient (1.1%) declared that they had abstained from taking any kind of preparation containing probiotic bacteria (Figure 3). Data from this patient were not included in the analysis of the present study. The remaining 90 patients (98.9%) declared that they had taken a pro- or synbiotic. Of these, 15 patients (16.7%) declared that they had taken other preparations apart from that evaluated in the ViIBS trial and were excluded from further analysis in the present study. The remaining 75 patients (83.3%) stated they had continued to take Vivatlac^®^ Synbiotic and were considered as the “treatment group” for the current study. Of the 75 patients who took Vivatlac^®^ Synbiotic during the 6 months until the follow-up examination, 10 patients reported an intake for 1–30 days, 45 for 31–90 days, and 20 for 91–180 days.

### 3.3. Baseline Characteristics of the Two Patient Groups

The patient baseline characteristics of the control group (*n* = 17) and the treatment group (*n* = 75) are summarized in Table 1. Statistical analyses revealed that there were no significant differences between the two groups with respect to weight, height, body mass index, and IBS questionnaire diagnosis score. Statistically significant differences (*p*-value ˂ 0.05) were found between the two groups for age, with patients in the treatment group being, on average, 5 years older than those in the control group. There were no statistically significant differences between the gender, IBS severity of disease, and IBS stool type distributions between the control and the treatment group.

### 3.4. Assessment of IBS Severity with the IBS-SSS

**Figure 4:**
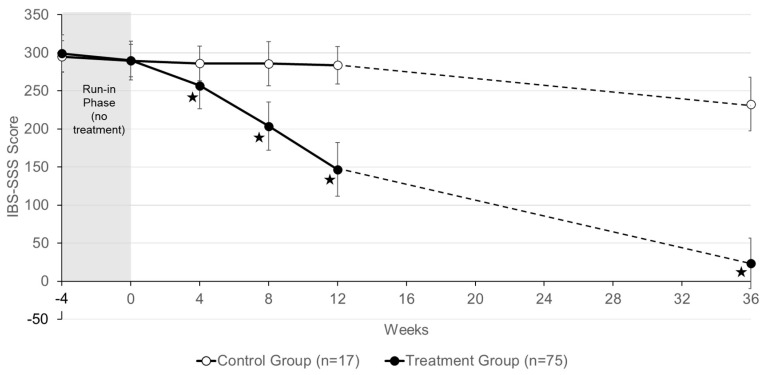
Change in IBS-SSS scores in patients of the control group and of the treatment group during the ViIBS trial (until week 12) and the 6 months of the observational study. Data shown are means ± S.D. The 4-week treatment-free run-in phase is highlighted in grey. Asterisks (★) indicate statistically significant differences (*p*-value ˂ 0.05) between the control group and the treatment group.

The results of assessing the severity of IBS symptoms with the IBS-SSS in patients participating in the follow-up examination are shown in Figure 4. Six months after finishing their trial participation, patients from the control group had an IBS score of 232.6 ± 35.1 (average ± S.D.) points, representing an average decrease of 51.1 points during the six-month period of the follow-up. In the treatment group, the IBS-SSS score of 146.8 ± 35.4 points at the end of the ViIBS trial decreased during the six-month follow-up period by an additional average of 123.3 points to a final value of 23.5 ± 33.1 points.

**Figure 5:**
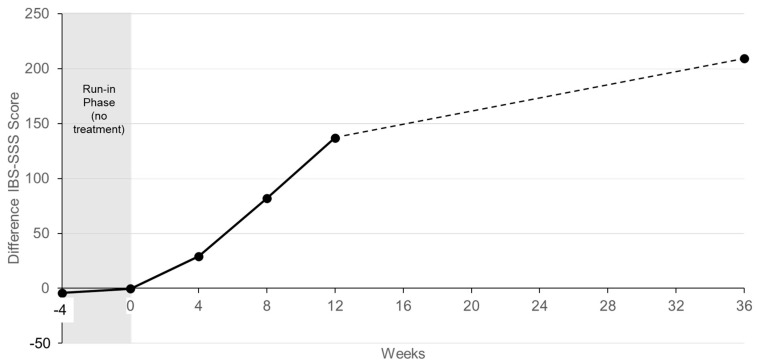
Change of the difference between average IBS-SSS scores of the treatment and the placebo group over time.

Figure 5 shows the differences between the average IBS-SSS scores of the treatment group and the control group as a function of time. As can be seen, the first four weeks of treatment with Vivatlac^®^ Synbiotic had a moderate effect (minus 33.1 points) on the severity of IBS symptoms. During the second and third month of treatment, the severity of symptoms decreased by 53.3 and 56.9 points, respectively. In the six months after the trial, the IBS-SSS score lowered by an additional 123.3 points, which, on a monthly basis, represents a decrease by about 20 points.

**Figure 6:**
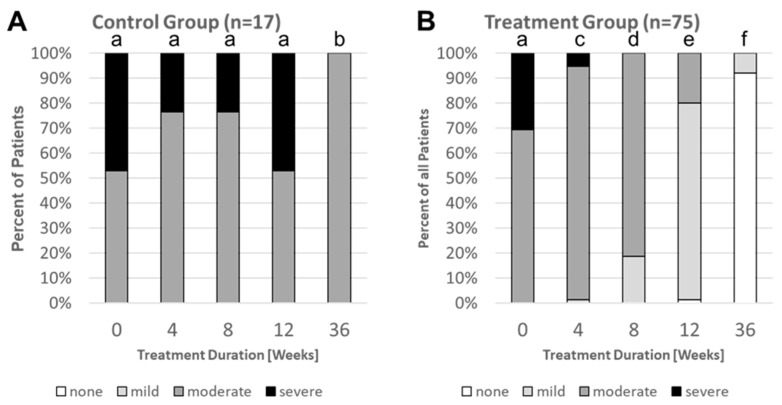
Changes in IBS severity (measured by using the IBS-SSS) over time in the control (**A**) and treatment (**B**) group. Different lowercase letters indicate significant differences between groups (*p*-value ˂ 0.05). For the control group, there were no significant differences between the IBS severity distribution at treatment durations of 0, 4, 8, and 12 weeks. The distribution determined after 36 weeks were significantly different (*p*-value ˂ 0.05) from those measured at earlier timepoints. The IBS severity distribution at 0 weeks in the treatment group was similar to that of the control group at 0 weeks. In the treatment group, the administration of the synbiotic resulted in a significant change (*p*-value ˂ 0.05) in the IBS severity distribution with every additional four weeks of treatment. Different lowercase letters indicate significant differences (*p*-value ˂ 0.05) between shown columns.

Individual IBS-SSS scores measured for each patient and time point were used to allocate the patient to one of four IBS severity categories (severe, moderate, mild, and none). For details, see Section 2. At the end of the ViIBS trial, the distribution of control-group patients between the severe and moderate IBS severity category had not changed compared to the distribution at enrollment (Figure 6A). During the following six months, all severe IBS cases of the control group had improved to the moderate IBS category.

At the end of the 12-week treatment, the administration of the synbiotic had resulted in the elimination of all severe cases and the transformation of the majority of the moderate cases into mild cases (Figure 6B). The administration of Vivatlac^®^ Synbiotic during the six months of the observational trial improved the IBS severity in most of the patients from the treatment group to the “none” IBS category (IBS-SSS score ˂ 75 points).

### 3.5. Diagnosis of IBS Using the IBS Questionnaire for HCPs by the WGO

**Figure 7:**
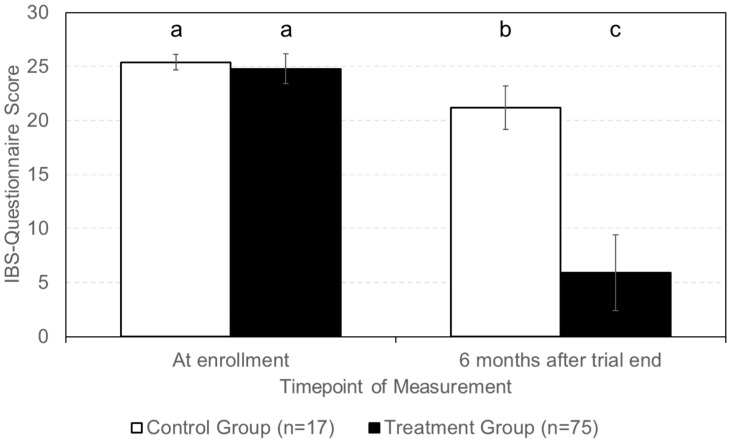
Scores determined with IBS questionnaire for HCPs of WGO at enrollment and six months after patients finalized their participation in the ViIBS trial. IBS questionnaire scores at enrollment for the control and the treatment group were not significantly different. Six months after the trial, IBS questionnaire scores for both the control and the treatment group significantly differed from their respective initial values (*p*-value ˂ 0.05). There was also a significant difference (*p*-value ˂ 0.05) between the control group score and that of the treatment group 6 months after the trial. Different lowercase letters indicate significant differences (*p*-value ˂ 0.05) between shown columns.

Diagnoses of IBS were performed at the enrollment of the ViIBS trial and at the end of the 6-month follow-up period by using the IBS questionnaire for HCPs of the WGO (Figure 7). The average IBS questionnaire scores at enrollment for the control-group and the treatment-group patients were 25.4 ± 0.7 and 24.8 ± 1.4, respectively. At the end of the follow-up period, the average score for patients from the control group was 21.2 ± 2.0, which was 4.2 points lower than at enrollment. For patients from the treatment group, the score changed from 24.8 ± 1.4 to 5.9 ± 2.5, a decrease of 18.9 points on average.

## 4. Discussion

### 4.1. Dropout Rates

Controlled clinical trials often have high dropout rates, sometimes exceeding 20% [26]. There are numerous factors that influence the attrition rate of a clinical trial, but particularly important ones are (i) the long trial duration, (ii) a patient demanding a strict study design with frequent follow-ups, (iii) the side effects of the intervention, or (iv) a lack of perceived benefit from the treatment [27]. In a recent review summarizing the clinical trials on the treatment effects of synbiotics in IBS patients, drop-out rates of up to 34% were found [15]. In the ViIBS trial, only 1 patient (0.5%) of the 202 patients participating in the study was lost [18]. The lost patient was from the placebo group and the reason for the absence at the last examination of the trial remains unclear. Of the 201 patients invited for a six-month follow-up examination, 161 attended, resulting in a dropout rate of 20%. Dropout rates were significantly different in the control group (30%) compared to the treatment group (10%). Patients in the treatment group of this study came from the ViIBS trial’s synbiotic treatment group and experienced significant IBS improvements compared to the placebo group. Conversely, patients in the control group, originating from the ViIBS trial’s placebo group, did not have this positive experience. While their motivation for participating in the follow-up examination was not investigated, the differing experiences of the placebo and synbiotic treatment groups during the ViIBS trial may explain the varying attrition rates observed in the two groups of the observational trial.

### 4.2. Patients’ Attitude Towards Intake of Products Containing Probiotic Bacteria

Of the 70 patients of the ViIBS trial placebo group who participated in the observational study, 53 took products containing probiotic bacteria and only 17 declared that they had abstained from taking this kind of product. A recently published study found that a recommendation by a doctor is a strong motivator for using pro- or synbiotics [28]. Although patients in the control group did not experience significant positive outcomes from their ViIBS trial participation, they might have been encouraged by information received from the physicians about the potential effects of this product category, before, during, and, especially, at the end of their participation.

Of the 101 patients from the synbiotic treatment arm of the ViIBS trial, 91 (90%) participated in the follow-up examination and 90 of these took a product containing probiotic bacteria. Among these 90, a total of 75 (83%) continued to take Vivatlac^®^ Synbiotic, the product evaluated in the active arm of the ViIBS trial. The low attrition rate observed and the relatively high adherence to the product tested in the ViIBS trial are most likely driven by the positive treatment effects experienced by patients from the synbiotic group during their participation in the ViIBS trial. Not all patients continued taking the trial product, likely because it is not commercially available in Poland and had to be ordered from an Internet pharmacy abroad.

### 4.3. Changes of IBS Symptoms Severity

The present analyses of the six-month follow-up data compared data from the ViIBS trial’s synbiotic treatment group who continued taking the products (*n* = 75) with those from the placebo group who declared that they had not taken products containing pro- or synbiotics (*n* = 17). While this analytical approach uses only parts of the follow-up data, it reveals differences in IBS symptom severity changes between a group of patients continuously taking the synbiotic evaluated in the ViIBS trial and those in a group not taking any probiotic products.

It would have been very interesting to evaluate the data of patients from the synbiotic treatment group of the ViIBS trial who discontinued taking the preparation as it would have made it possible to determine if the treatment effects of the synbiotic would have been sustained or faded after stopping the intake. Unfortunately, there was only one patient from the synbiotic treatment group who declared that they had not taken a product containing probiotic bacteria during the six-month follow-up period. Consequently, this patient category could not be analyzed in the present study due to the lack of sufficient data.

Patients taking products containing probiotic bacteria other than that evaluated in the ViIBS trial were excluded from the present analysis as a large variety of different products were consumed by patients, resulting in a large number of data sets, each with very few patients taking a particular product.

During the six months after the ViIBS trial, patients in the treatment group who continued taking the synbiotic saw their IBS-SSS score drop by an additional 123.3 points, resulting in a final average score of 23.5 points. In contrast, the control group had a decrease of only 51.1 points, ending with a final score of 232.6 points. The monthly reduction in IBS symptom severity for the treatment group was 20 points, lower than the reductions observed during the second (53.3 points) and third (56.9 points) months of the trial, likely because patients had already experienced significant improvements.

Patients reported their synbiotic intake over the six months as low (1–30 days), medium (31–90 days), or high (91–180 days), but the analysis showed no significant differences in symptom severity reduction among these groups. The interpretation of this observation should be cautious, as it was based purely on patient feedback without the support of tools like diaries, which might not be very accurate. If there is no difference between the dosage groups, it suggests daily intake may not be necessary to achieve the observed effect; intermittent administration might suffice. However, without more frequent measurements, we cannot rule out the possibility that regular daily intake might bring about symptom improvement sooner.

The reason for the symptom improvement in the control group remains unclear, but spontaneous IBS symptom improvements have been noted in other clinical trials [29,30].

### 4.4. Diagnosis of IBS with the IBS Questionnaire for HCPs

The IBS questionnaire for health care professionals was used for the diagnosis of patients during the enrollment examination of the ViIBS trial. During the six-month follow-up examination, the questionnaire was used again to repeat the patient diagnosis for IBS. The average IBS questionnaire scores were 5.9 and 21.2 for the treatment group and the control group, respectively. For patients with IBS-questionnaire scores below 15, it is assumed that their symptoms may not be due to IBS but due to other conditions [19]. Patients with scores ranging from 15 to 24 points on the IBS questionnaire may suffer from IBS but other conditions, which have to be excluded by the diagnosing physician, are also possible. The average score of the treatment group indicates that these patients are no longer diagnosed as IBS patients. This corresponds with the average IBS-SSS score determined for this patient group, which indicates the absence of IBS symptoms. In contrast, the average IBS questionnaire score of control patients is at a level which still corresponds to being diagnosed as IBS patients, which is in line with these patients being categorized as moderate IBS patients based on their IBS-SSS scores.

Both the IBS-SSS and the IBS questionnaire measurements in patients from the treatment group hint that extending the intake of the synbiotic after the 12-week treatment during the ViIBS trial by an additional six months has the potential to decrease the severity of IBS symptoms to a level where patients would no longer be diagnosed as IBS patients. No such effect was observed in the control group consisting of patients who abstained from taking products containing probiotic bacteria.

### 4.5. Strengths of the Study

Being an observational trial, the current study has significant limitations and the results observed should be interpreted with great caution. However, the study provided a number of interesting and important insights which will be of great help in planning a randomized, double-blind, placebo-controlled follow-up trial of the ViIBS trial.

Among the strengths of the study is the fact that data from 161 patients (80% of all ViIBS trial participants) could be collected. This number is higher than the typical patient count (often less than 100) in clinical IBS trials investigating probiotic products published so far [11,12,13,14,15]. The two patient groups examined for the present study were large enough to allow some meaningful analyses. While the observed treatment effect of the continued administration of the synbiotic has to be interpreted with care, the observed impact is at least encouraging for performing a follow-up study of the ViIBS trial. In addition, the attrition rates determined provide some idea for the sample size calculation of a future trial.

### 4.6. Limitations of the Study

Being an observational study, the limitations of the present study are numerous. Data from the present study originate from one single examination performed six months after patients had finished their participation in the ViIBS trial. More frequent measurements would have made it possible to establish a better understanding of the time development of the observed effects but this had to be omitted to limit the workload for doctors and patients. The present study followed an open-label design with patients being fully aware of what preparation, if any, they were taking. The positive experiences of the majority of patients in the ViIBS trial’s treatment group may have led to a positive bias regarding the effects of the synbiotic. A very simple approach for determining the number of daily doses taken by patients was employed and data from these measurements might not be very reliable. Patients who declared that they had abstained from products containing probiotic bacteria might have unconsciously consumed this kind of product, e.g., in the form of dairy products. A large proportion of the patients from the placebo group of the ViIBS trial (53 of 100) started taking products containing probiotic bacteria after their trial participation. Of these, nearly half (*n* = 25) started taking the synbiotic evaluated in the ViIBS trial. While this speaks for the tested preparation, it might have resulted in a control group composition (those patients abstaining from taking pro- or synbiotics) which might have a particular characteristic influencing the results. In addition, it resulted in a significant size difference in the treatment group (*n* = 75) compared to the control group (*n* = 17). A deviation from a 50/50 treatment and control split, as observed in the present study, harms the statistical power which has to be considered as a significant limitation when comparing the results obtained for the treatment group with those of the control group. Only results of a high-quality randomized, double-blind and placebo-controlled clinical trial will allow the treatment effects caused by the long-term administration of the synbiotic in comparison to a placebo intake to be judged. Such a trial is currently in the planning.

## 5. Conclusions

Due to the design of the study (cross-sectional observational study with only one examination), the results of the present study have to be interpreted with care. However, the observed motivation of patients to continue the administration of the synbiotic preparation and its observed treatment effects are very encouraging to further investigate this product in future randomized, double-blind, placebo-controlled clinical trials. If these trials confirm that the prolonged intake of the preparation reduces the IBS severity of symptoms to levels similar to those observed in the present observational study, it could greatly impact the future management of IBS patients. A potential ViIBS II trial should investigate the effects of the balanced multi-strain synbiotic in a multi-center, randomized, double-blind and placebo-controlled trial with a treatment duration of nine months. For the sample size calculation, it has to be considered that the attrition rates might be in the range of 20% for the treatment group and 30% or higher for the placebo group.

## Figures and Tables

**Table 1:** Patient baseline characteristics of control and treatment group assessed by study doctors during the enrollment examination of patients of the ViIBS trial and by analyzing patients’ self-reporting during the run-in phase of the ViIBS trial. Data shown are means ± S.D., or absolute numbers. Percentage values indicate size of group in percent of total number of patients in the respective group. Statistically significant differences are indicated by *p*-values ˂ 0.05.

	Control Group (*n* = 17)	Treatment Group (*n* = 75)	*p*-Value
Weight (kg)	69.6 ± 11.0	71.0 ± 12.6	0.6687 ^a^
Height (cm)	171.2 ± 8.7.0	171.7 ± 9.5	0.9114 ^a^
Body Mass Index (kg/m^2^)	23.6 ± 2.0	23.9 ± 2.1	0.5663 ^a^
Age (years)	38.2 ± 8.4	43.2 ± 9.1	0.0372 ^a^
Gender (female/male)	11/6 (65%/35%)	47/28 (63%/37%)	0.8750 ^b^
IBS questionnaire for HCPs (score)	25.4 ± 0.7	24.8 ± 1.4	0.1294 ^a^
IBS-SSS Severity (moderate/severe)	10/7 (59%/41%)	41/34 (55%/45%)	0.7555 ^b^
IBS-SSS Stool Type (IBS-D/IBS-C/IBS-M/IBS-U) ^c^	10/3/0/0 (82%/18%/0%/0%)	50/24/0/1 (67%/32%/0%/1%)	0.4296 ^b^

^a^ Mann–Whitney U-test, ^b^ Pearson’s Chi-squared test, ^c^ IBS-D: IBS with diarrhea, IBS-C: IBS with constipation; IBS-M: IBS with diarrhea and constipation, IBS-U: unspecified IBS.

## Data Availability

The data presented in this study are available upon reasonable request from the corresponding author due to ethical reasons.
